# Sexually Transmitted Dermatophytes Can Cause Severe Infection Among Men who Have Sex With Men as Tinea Genitalis

**DOI:** 10.1093/ofid/ofad519

**Published:** 2023-11-17

**Authors:** David Chromy, Anthea-Margaux Osmers, Wolfgang Michael Bauer, Veronique Touzeau-Roemer, Carina Borst, Stefan Esser, Wolfgang Weninger, Birgit Willinger, Katharina Grabmeier-Pfistershammer

**Affiliations:** Department of Dermatology, Medical University of Vienna, Vienna, Austria; Department of Dermatology and Venereology, University Hospital Essen, University Duisburg-Essen, Essen, Germany; Department of Dermatology, Medical University of Vienna, Vienna, Austria; Department of Dermatology, Medical University of Vienna, Vienna, Austria; Department of Dermatology, Medical University of Vienna, Vienna, Austria; Department of Dermatology, Medical University of Vienna, Vienna, Austria; Department of Dermatology and Venereology, University Hospital Essen, University Duisburg-Essen, Essen, Germany; Department of Dermatology, Medical University of Vienna, Vienna, Austria; Division of Clinical Microbiology, Department of Laboratory Medicine, Medical University of Vienna, Vienna, Austria; Department of Dermatology, Medical University of Vienna, Vienna, Austria

**Keywords:** HIV, men who have sex with men, PrEP, tinea genitalis

## Abstract

Very limited data on tinea genitalis, a potentially severe dermatophytosis transmitted during sexual intercourse affecting the genital area, suggest its potential to cause outbreaks. Thus, we investigated genital dermatophyte infections at an HIV/sexually transmitted infection clinic and identified 17 men who have sex with men (all people with HIV or pre-exposure prophylaxis users) diagnosed with tinea genitalis.

Cumulative data demonstrate that men who have sex with men (MSM) are more than proportionally affected by sexually transmitted infections (STIs) [1]. Prominent examples are the HIV epidemic [2], hepatitis C virus infections [3], lymphogranuloma venereum [4], and syphilis [5] among MSM. Furthermore, since the advent of HIV pre-exposure prophylaxis (PrEP), a surge of “classical” STIs among HIV-negative MSM has been described, including gonorrhea, chlamydia, and *Mycoplasma genitalium* infections [1, 6]. Additionally, several reports indicate increased rates of other infectious diseases among MSM that are commonly transmitted by smear infection, like the 2022 Mpox outbreak [7], or by the fecal–oral route like hepatitis A virus [8] and enteric infections (eg, *Shigella*, *Campylobacter*) [9]. Another infection that is commonly transmitted during close contact is tinea, also known as “ringworm” [10]. Typically caused by dermatophytes like *Trichophyton* spp. or *Microsporum* spp., this disease can cause superficial infections of the skin or may present as a deep invasive infection requiring prolonged systemic treatment in both immunocompromised and immunocompetent individuals [10]. A 2015 report on tinea in the genital area suggested transmission of dermatophytes during sexual intercourse, which, mechanistically, would be plausible [11]. Yet, data on dermatophytic infections in MSM—a population clearly vulnerable to sporadic infections—are currently limited to a single report describing a series of *Trichophyton metagrophytes* infections among MSM in France [12]. We thus aimed to investigate all infections with dermatophytes at a large HIV/STI clinic and the linkage to sexual activity and transmission groups.

## METHODS

All mycologic test results (culture and polymerase chain reaction [PCR]–based tests) at the HIV/STI clinic of the Medical University of Vienna between January 2014 and March 2022 were retrospectively reviewed. While the HIV/STI clinic is a tertiary care facility, it is also open for acute and individual appointments, comparable to a walk-in clinic concept. Patients with positive results for dermatophyte infections were then identified, and further details on the patient's characteristics, course of disease, and potential mode of transmission were collected from the medical records. All yeast infections (eg, intertrigo, *Candida* vulvovaginitis, *Candida* stomatitis) and “undetermined” (culture only) mycologic results were not further considered. Linkage to sexual intercourse was defined as (i) sexual intercourse ≤12 weeks before the onset of symptoms AND (ii) reported sex practices including the affected region AND (iii) absence of other probable transmission vectors. Tinea genitalis was defined as a tinea-typical skin lesion, a positive test for any dermatophyte, and meeting the criteria for “linkage to sexual intercourse.” Descriptive statistics were performed using GraphPad Prism 9 (GraphPad Software, La Jolla, CA, USA).

## RESULTS

**Figure 1.**
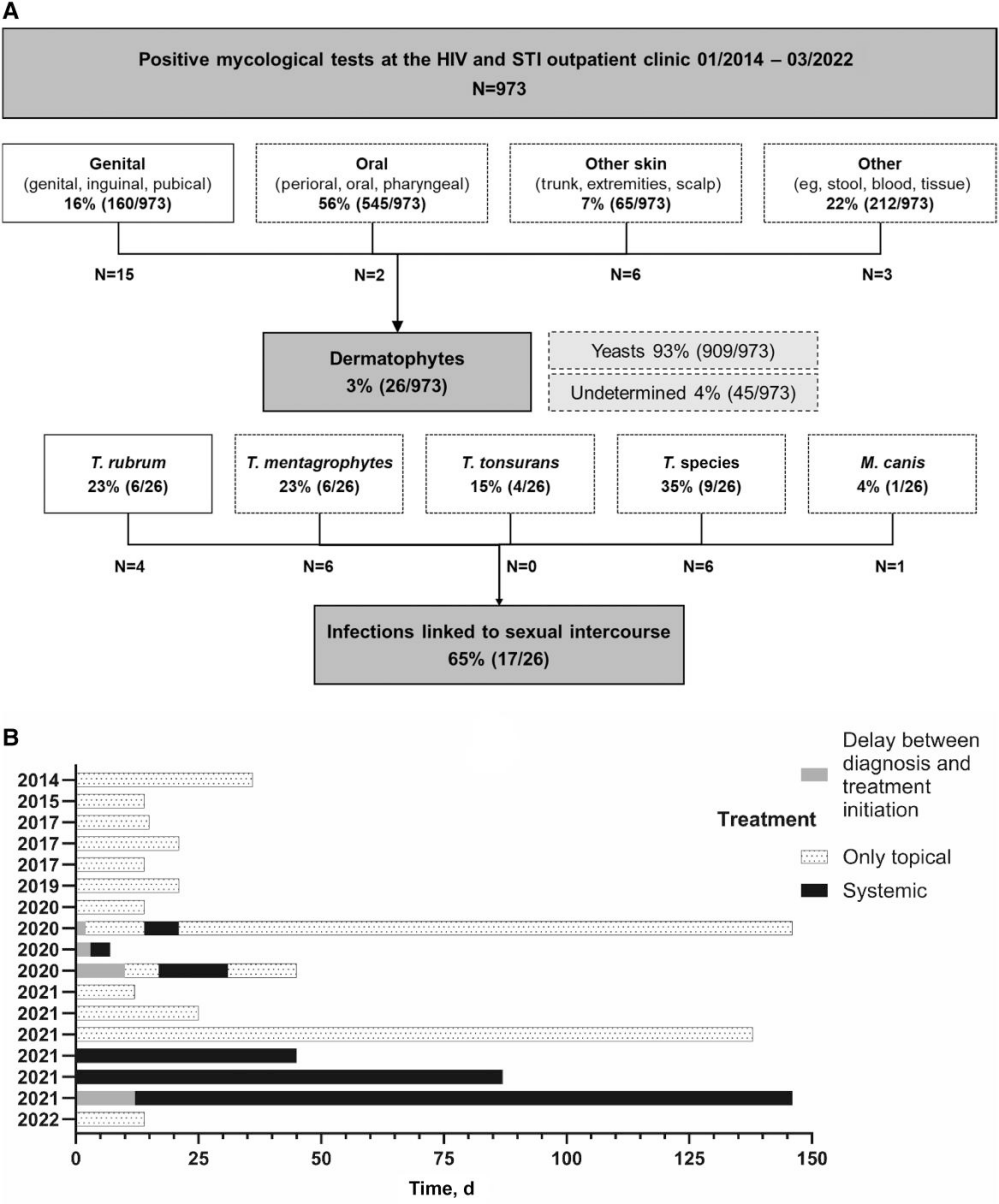
*A*, Flowchart. *B*, Treatment course of individual patients. Each timeline denotes the duration of every single patient’s treatment. The number corresponds to each case described in the table. Abbreviation: STI, sexually transmitted infection.

We identified 973 positive mycological tests obtained at our HIV/STI clinic during the investigational period (57% [556/973] male: 35% [192/556] MSM, of which 9% [18/192] were on PrEP; 55% [540/973] HIV+: 32% [174/540] MSM). The vast majority (93% [909/973]) were yeast infections, whereas dermatophytes accounted for only 3% (26/973) of all fungal infections (Figure 1*A*). Of the latter, all but 1 (96% [25/26]) were caused by *Trichophyton* species; only 1 *Microsporum canis* infection was detected. Nine dermatophyte infections were not linked to sexual intercourse: all 4 *T. tonsurans* infections (n = 3 women, n = 1 man) affected the trunk and were either transmitted during sports (n = 2) or remained undetermined (n = 2); 3 cases of tinea capitis (n = 2 children, n = 1 heterosexual man); 1 case of *T. interdigitale* and *T. mentagrophytes* in a heterosexual man and an MSM with HIV, respectively, both found on the trunk after recent animal contact. All remaining dermatophyte infections matched the criteria for linkage to sexual intercourse and were found in MSM (100% [17/17]). Thirty-five percent (6/17) of the affected MSM were using PrEP, and all others (65% [11/17]) were people with HIV (PWH) successfully treated with antiretroviral therapy (Table 1), with a median CD4^+^ cell count (range) of 721 (403–1310) per μL. None of the patients received immunosuppressive treatment or had any other condition causing a decrease in immune function. Upon presentation, 9 patients reported predominantly pain in conjunction with the skin lesion. While some patients experienced mild symptoms with few small plaques, others showed highly inflammatory lesions with extensive plaques and pustules. Concomitant infections with syphilis, gonorrhea, and chlamydia were found in 12% (2/17), 18% (3/17), and 12% (2/17), respectively. More than half of all cases were detected within only 2 years between January 2020 and March 2022 (59% [10/17]), and *T. mentagrophytes* (n = 6) occurred exclusively during this period. All patients received topical treatment, which was usually initiated upon clinical presentation. However, in 4 cases, treatment was not started until the microbiological results were available (Figure 1*B*). Isoconazole (n = 9) and terbinafine (n = 5) were the most common regimens for topical treatment. Four individuals received fluconazole, and 2 individuals received terbinafine systemically, while hospitalization was required in 1 case for further management. The median treatment duration (including topical and systemic treatment) was 21 days; however, the range was 4–144 days, and all patients achieved disease resolution. Of note, all treatment regimens were chosen empirically, and resistance analyses for dermatophytes were not routinely performed and, thus, not available for any case of tinea genitalis.

**Table 1.** Characteristics of all Individuals Diagnosed With Tinea Genitalis

	Epidemiologic Characteristics			Infection-Specific Characteristics								
Case	Age	HIV	PrEP	Year	Location	Pathogen	Symptoms	Clinical Presentation	Topical Treatment	Systemic Treatment	Hospitalized	Concomitant Infections
1	38	Yes, on ART	No	2014	Penis	*T. rubrum*	Pain and pruritus	Papules and pustules	Isoconazole	-	-	Syphilis
2	48	Yes, on ART	No	2015	Gluteal	*M. canis*	Pruritus	Papules and pustules	Isoconazole	-	-	…
3	57	Yes, on ART	No	2017	Inguinal	*T.* species^a^	Pain	Papules	Isoconazole	-	-	-
4	59	Yes, on ART	No	2017	Perianal	*T.* species^a^	None	Papules	Isoconazole	-	-	Syphilis
5	28	No	Yes	2017	Penis	*T.* species^a^	None	Papules and pustules	Ketoconazole	-	-	Gonorrhea
6	39	Yes, on ART	No	2019	Perianal	*T.* species^a^	None	Papules	Clotrimazole	-	-	-
7	41	Yes, on ART	No	2020	Penis	*T. mentagrophytes*	Pain	Papules	Isoconazole	-	-	Gonorrhea
8	39	Yes, on ART	No	2020	Pubic	*T. mentagrophytes*	Pruritus	Papules	Isoconazole	Fluconazole	-	Syphilis
9	41	Yes, on ART	No	2020	Pubic	*T.* species^a^	Pain and pruritus	Papules, pustules, and abscesses	Terbinafine	Terbinafine	7 d	-
10	28	No	Yes	2020	Scrotum	*T. rubrum*	Pain and pruritus	Papules and pustules	Isoconazole	Fluconazole	-	-
11	32	Yes, on ART	No	2021	Gluteal	*T. mentagrophytes*	Pain and pruritus	Papules	Isoconazole	-	-	Gonorrhea and chlamydia
12	40	No	Yes	2021	Perioral and cheek	*T. mentagrophytes*	None	Papules and pustules	Terbinafine	-	-	Chlamydia
13	33	No	Yes	2021	Pubic	*T. mentagrophytes*	Pruritus	Papules	Terbinafine	-	-	-
14	32	No	Yes	2021	Gluteal	*T.* species^a^	Pain	Papules and pustules	Terbinafine	Fluconazole	-	-
15	31	No	Yes	2021	Perianal and gluteal	*T. rubrum*	None	Papules	Terbinafine	Terbinafine	-	-
16	33	Yes, on ART	No	2021	Perioral and lips	*T. rubrum*	Pain	Papules and pustules	Terbinafine	Fluconazole	-	-
17	33	Yes, on ART	No	2022	Inguinal	*T. mentagrophytes*	Pain	Papules, pustules, and abscesses	Isoconazole	-	-	-

Abbreviations: ART, antiretroviral therapy; MSM, men who have sex with men; PrEP, pre-exposure prophylaxis for HIV.

^a^In a limited number of cases, the subtype analysis for *Trichophyton* was not performed; thus, details were unavailable.

## DISCUSSION

Our work is one of the very few reports suggesting sexual transmission of dermatophyte infections as tinea genitalis. Thus, it adds to the cumulative evidence that it is its own disease entity and an infection transmissible by sexual intercourse. Dermatophytes can be transmitted by a variety of vectors, yet we believe that “tinea genitalis” is an appropriate term for infections linked to sexual intercourse—comparable to the association of “tinea gladiatorum” with wrestling. Furthermore, in our analysis, all affected individuals were MSM, and the majority of infections occurred within a relatively short time frame. The first report on tinea genitalis was published by Luchsinger and coworkers in 2015 and described a case series of 7 European travelers returning with inflammatory tinea in the genital region after they had sexual intercourse with a local person in Southeast Asia [11]. The authors pointed out that the diagnosis might be challenging to differentiate from eczema or folliculitis, yet early recognition and treatment initiation are incremental for complete disease resolution. In 2018, Kupsch and coworkers published a collection of 43 patients with suspected tinea genitalis, and *Trichophyton* spp. were detected in all but 4 patients [13]. Interestingly, the authors could demonstrate that a specific genotype of *T. mentagrophytes* accounted for the majority of cases, and isolates from 30 patients were subjected to phylogenetic analysis, enabling the authors to demonstrate a connection between those cases that was suggestive of an outbreak [13]. Unfortunately, this study did not provide further information on the risk group allocation of the individuals. Our study analyzed all fungal infections at our HIV/STI clinic throughout the study period and identified 17 cases exclusively affecting MSM. The HIV/STI clinic is attended by a broad spectrum of individuals (eg, 43% of mycologic tests were obtained from women) with various symptoms and conditions in the genital area. We can thus only hypothesize about the reasons for having observed tinea genitalis only among MSM. All affected MSM in our study were either PWH or PrEP users, which are known predictors for casual dating associated with a comparatively high number of sex partners as well as prolonged intercourse facilitated by the use of phosphodiesterase-5 inhibitors and/or recreational drugs [6, 14]. These behavioral patterns are likely favorable for both the person-to-person transmission and creating a transmission network of tinea genitalis. This assumption is further supported by a very recent report on 13 cases of sexually transmitted tinea affecting almost exclusively MSM (92% [12/13]) [12] as well as the recent Mpox outbreak that additionally highlighted this population’s vulnerability to an epidemic [7]. However, a recent case report of a heterosexual man presenting with tinea genitalis after unprotected sexual intercourse with several women shows that this disease can affect various demographic populations [15].

A strong aspect of our work is the long observational period and the comprehensive characterization of the individuals diagnosed with tinea genitalis in conjunction with details on treatment and outcome. Nonetheless, the retrospective design presents a major limitation of our work, potentially introducing a variety of biases. Due to the retrospective data collection, we have no details on the patients’ shaving status available—a factor previously described as a promotor of tinea genitalis manifestation [11]—and susceptibility analyses of the detected dermatophytes were not performed for the following practical reasons: Until recently, there has been no reason to doubt the efficacy of empiric treatment of dermatophytes with terbinafine or azoles. Additionally, most (15/17) of the tinea genitalis cases were diagnosed using PCR only, allowing fast treatment initiation and early clinical evaluation of the treatment response—diagnosis by culture and consecutive susceptibility testing would have taken multiple weeks. Lastly, we were unable to provide a phylogenetic analysis that could have added details on transmission clusters.

In conclusion, growing evidence shows that “tinea genitalis” represents a distinct dermatophytosis transmitted during sexual intercourse. We need future studies that address details on the transmission group and phylogenetic cluster analysis to better understand the transmission networks and whether a specific variant of *Trichophyton* favors manifestation as tinea genitalis. Importantly, as tinea genitalis can manifest as a severe and potentially scarring infection, it should be considered in all individuals presenting with skin lesions in the genital area.
